# Chitosan alleviates ovarian aging by enhancing macrophage phagocyte-mediated tissue homeostasis

**DOI:** 10.1186/s12979-024-00412-9

**Published:** 2024-01-27

**Authors:** Hui-Hui Shen, Xin-Yan Zhang, Nan Liu, Yang-Yang Zhang, Hui-Hua Wu, Feng Xie, Wen-Jun Wang, Ming-Qing Li

**Affiliations:** 1 Laboratory for Reproductive Immunology, Hospital of Obstetrics and Gynecology, Fudan University, Shanghai, 200080 People’s Republic of China; 2 Institute of Obstetrics and Gynecology, Hospital of Obstetrics and Gynecology, Fudan University, Shanghai, 200080 People’s Republic of China; 3 Shanghai Medical College, Fudan University, Shanghai, 200032 People’s Republic of China; 4 Center of Reproduction and Genetics, The Affiliated Suzhou Hospital of Nanjing Medical University, Suzhou Municipal Hospital, Gusu School, Nanjing Medical University, Suzhou, 215002 People’s Republic of China; 5 Department of Gynecology of Integrated Traditional Chinese and Western Medicine, Hospital of Obstetrics and Gynecology, Fudan University, Shanghai, 200011 People’s Republic of China; 6 Shanghai Key Laboratory of Female Reproductive Endocrine Related Diseases, Hospital of Obstetrics and Gynecology, Fudan University, Shanghai, 200080 People’s Republic of China

**Keywords:** Ovary, Macrophages, Aging; phagocytosis, Chitosan, Diminished ovarian reserve

## Abstract

**Background:**

Age-related changes in the ovarian microenvironment are linked to impaired fertility in women. Macrophages play important roles in ovarian tissue homeostasis and immune surveillance. However, the impact of aging on ovarian macrophage function and ovarian homeostasis remains poorly understood.

**Methods:**

Senescence-associated beta-galactosidase staining, immunohistochemistry, and TUNEL staining were used to assess senescence and apoptosis, respectively. Flow cytometry was employed to evaluate mitochondrial membrane potential (MMP) and apoptosis in granulosa cells lines (KGN), and macrophages phagocytosis. After a 2-month treatment with low molecular weight Chitosan (LMWC), ovarian tissues from mice were collected for comprehensive analysis.

**Results:**

Compared with the liver and uterus, the ovary displayed accelerated aging in an age-dependent manner, which was accompanied by elevated levels of inflammatory factors and apoptotic cells, and impaired macrophage phagocytic activity. The aged KGN cells exhibited elevated reactive oxygen species (ROS) and apoptotic levels alongside decreased MMP. H_2_O_2_-induced aging macrophages showed reduced phagocytosis function. Moreover, there were excessive aging macrophages with impaired phagocytosis in the follicular fluid of patients with diminished ovarian reserve (DOR). Notably, LMWC administration alleviated ovarian aging by enhancing macrophage phagocytosis and promoting tissue homeostasis.

**Conclusions:**

Aging ovarian is characterized by an accumulation of aging and apoptotic granulosa cells, an inflammatory response and macrophage phagocytosis dysfunction. In turn, impaired phagocytosis of macrophage contributes to insufficient clearance of aging and apoptotic granulosa cells and the increased risk of DOR. Additionally, LMWC emerges as a potential therapeutic strategy for age-related ovarian dysfunction.

**Supplementary Information:**

The online version contains supplementary material available at 10.1186/s12979-024-00412-9.

## Background

The ovary commands considerable attention within the scope of aging research, owing to its discernible vulnerability to the passage of time. Among human females, the ovary presents a marked display of age-associated phenomena: a steady decline in reproductive capacity becomes evident in the mid-30 s, ultimately culminating in menopause during the late 40 s to 50 s. As women transition into their early thirties, a precipitous decline in fertility becomes apparent, accompanied by a rapid escalation in the likelihood of miscarriages and pregnancy complications [1]. This is also exacerbated by a notably reduced success rate in the realm of *in-vitro* fertilization (IVF) procedures. Among individuals diagnosed with diminished ovarian reserve (DOR), younger women exhibit more favorable reproductive outcomes through the application of assisted reproductive technology when compared with their older counterparts [2–5].

As life expectancy continues to rise, a growing number of women confronts tough challenges in the realm of reproduction as they advance into their later years. A recent investigation, grounded in alterations discerned in transcripts, proteins, metabolites and other dimensions across a cohort of 113 healthy individuals, has identified two inflection points of aging in women-30 and 50 years of age. In terms of serum hormones, follicle-stimulating hormone (FSH) and luteinizing hormone (LH) manifest as the most positively correlated with age, while anti-Müllerian hormone (AMH) exhibits the most prominent downregulation [6]. A previous study has also identified the pivotal age of 34 as a critical temporal juncture on the aging clock. More specifically, aging-related biological pathways and proteins exhibit a high degree of conservation between mice and humans, including GDF15 and IGF1, illuminating a pivotal transition point of aging through alterations in peripheral blood constituents [7]. However, the precise mechanisms underlying fertility decline at 34 years old remain elusive. In addition, prior investigations have predominantly centered upon delineating the impact of aging on oocytes [8–11]. The changes occurring within the ovarian microenvironment during the aging process, remain largely unknown.

Ovarian microenvironment, which serves as the nurturing milieu for oocyte maturation, experiences profound alterations due to the aging process. The immune system is a critical component of the ovarian microenvironment, playing pivotal roles in a myriad of physiological processes within the ovary. These roles range from orchestrating follicle development and facilitating ovulation, to participating in luteal formation and regression [12–14]. Among them, macrophages hold a prominent position within the mammalian ovary. They are active participants in processes such as inflammation and phagocytosis-a process of ingesting a variety of cellular substrates, mainly bacteria and cellular debris [15]. Additionally, macrophages display significant plasticity, finely tuned to their tissue environment [16]. As the necessity to clear accumulated atretic follicles grows, it is important to unravel the interplay between aging and the ovarian immune milieu.

Therefore, the aim of our study was to investigate the characterization of aging ovarian and the crosstalk between macrophage and granulosa cells (GCs) in aging ovarian microenvironment, further explore the potential therapeutic strategy.

## Results

### Ovary undergoes aging at an earlier stage compared with the liver and uterus

Aging is a complex biological process that affects different organs and tissues in a distinct manner. To discern potential disparities in age-related changes, our investigation embarked on discerning the aging patterns of two distinct systems: the female reproductive system, represented by the ovary and uterus, and the digestive system, represented by the liver. Ovaries, uterus, and livers were collected from C57BL/6 mice at 3-month, 6 months and 9 months of age.

**Fig. 1:**
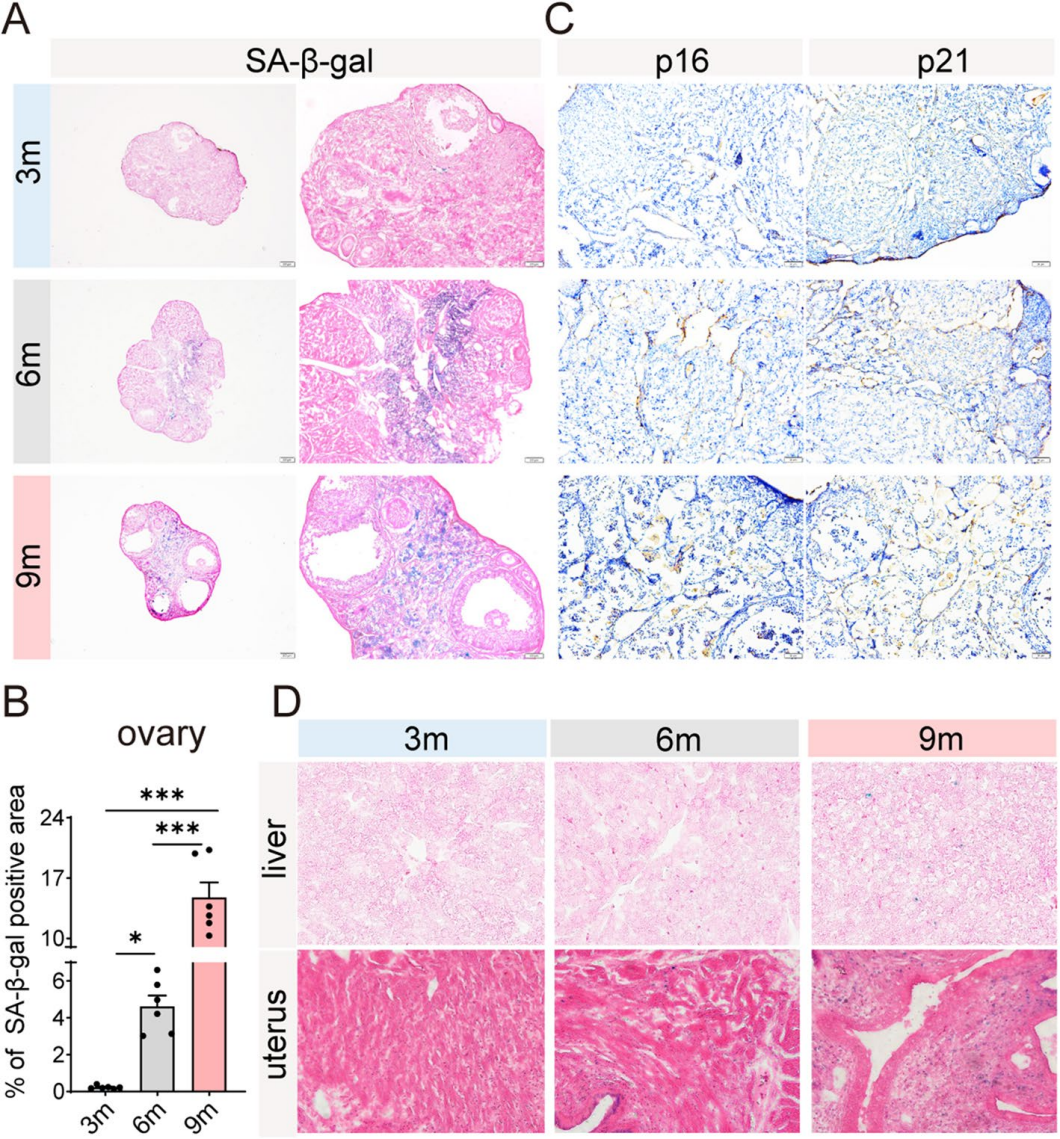
Ovary undergoes aging at an earlier stage compared to the liver and uterus. **A** Representative images of senescence-associated β-galactosidase (SA-β-gal) staining in murine ovarian sections, with corresponding scale bars provided for each image. **B** Quantitative assessment of the SA-β-gal-positive area in ovarian tissues from 3-month (3 m), 6-month (6 m), and 9-month (9 m) mice (*n* = 6 for each group). *P < 0.05, **P < 0.01, ***P < 0.001. **C** Representative immunohistochemical images showing the expression of p16 and p21 in murine ovarian tissues. **D** Representative SA-β-gal staining images in the uterus and liver sections of mice at 200 × magnification

As illustrated in Fig. 1A, the minimal SA-β-gal activity, a well-established marker of cellular senescence [17], was detected in the ovaries of 3- and 6-month-old mice, while a marked increase was observed in the ovaries of 9-month-old mice (Fig. 1B). Additionally, the expression of two pivotal cell cycle suppressor proteins, p16 and p21 [18], were elevated at 6 months and persisting through 9 months of age (Fig. 1C). Conversely, the liver and uterus of 9 months of age, exhibited a limited presence of SA-β-gal-positive cells (Fig. 1D). Apparently, the ovaries exhibited an early and higher accumulation of senescent cells during murine aging.

### Age-associated decline in ovarian function correlates with elevated expression of inflammatory factors

**Fig. 2:**
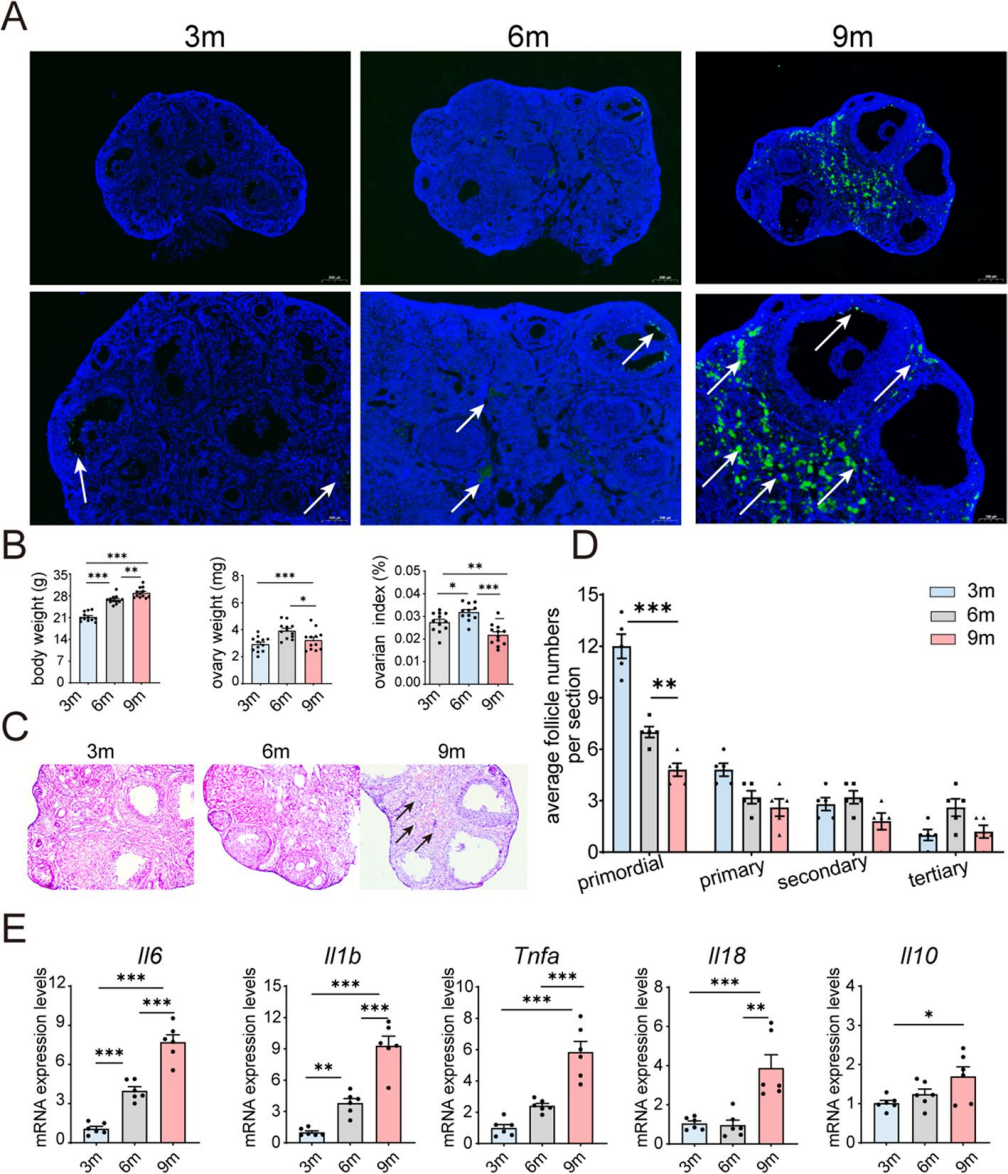
Age-associated decline in ovarian function correlates with elevated expression of inflammatory factors. **A** Visualization of TUNEL-labeled apoptotic cells (in green) with DAPI counterstaining (in blue) in the ovaries of mice at 3 months (3 m), 6 months (6 m), and 9 months (9 m) of age. **B** Comprehensive data on body weight, ovary weight, and ovarian index for each group of mice (*n* = 12 for each group). **C** Representative histological images obtained through H&E staining of murine ovarian tissue sections from each experimental group (at 200 × magnification). Atretic follicles are indicated by black arrows. **D** Quantitative analysis of the average number of follicles per tissue section for each experimental group (*n* = 5 for each group). **E** Assessment of relative mRNA expression levels for genes associated with inflammation and senescence-associated secretory phenotype (SASP) (*n* = 6 for each group). *P < 0.05, **P < 0.01, ***P < 0.001

Aging is charactered by the gradual decline in ovary function. In this study, a substantial increase of TUNEL-positive cells was observed with aging in the ovary of 9-month-old mice, predominantly localized in the ovarian stromal and medullary areas (Fig. 2A), which echoed the areas of SA-β-gal positive staining. Furthermore, the body weight of the three groups of mice increased steadily. Differently, the ovarian weight increased at 6 months but declined at 9 months when compared to the 3-month group, and the ovarian index exhibited a marked decrease at the 9-month (Fig. 2B).

Ovaries of 3-month-old mice contained abundant numbers of primordial follicles (Fig. 2C). However, at 6 months of age, the number of primordial follicles decreased to approximately 50% of that observed at 3 months, with the most significant decline observed at 9 months (Fig. 2C-D), suggesting a diminish in ovarian reserve during ovarian aging. By histological examination, we observed an increased incidence of atretic follicles and infiltration with inflammatory cells in the 9-month group (Fig. 2D).

The accumulation of senescent cells within ovary is likely to contribute to age-related chronic inflammation [19, 20]. In our study, RT-PCR analysis revealed a significant increased expression of inflammatory molecules (*Il6, Il1β, Tnfa, Il18, Il10*) in the ovaries of mice, revealing a positive correlation with aging (Fig. 2E). This pro-inflammatory environment should be a significant factor for age-related alterations in ovarian function.

### Enhanced oxidative stress and apoptosis in aging granulosa cells

**Fig. 3:**
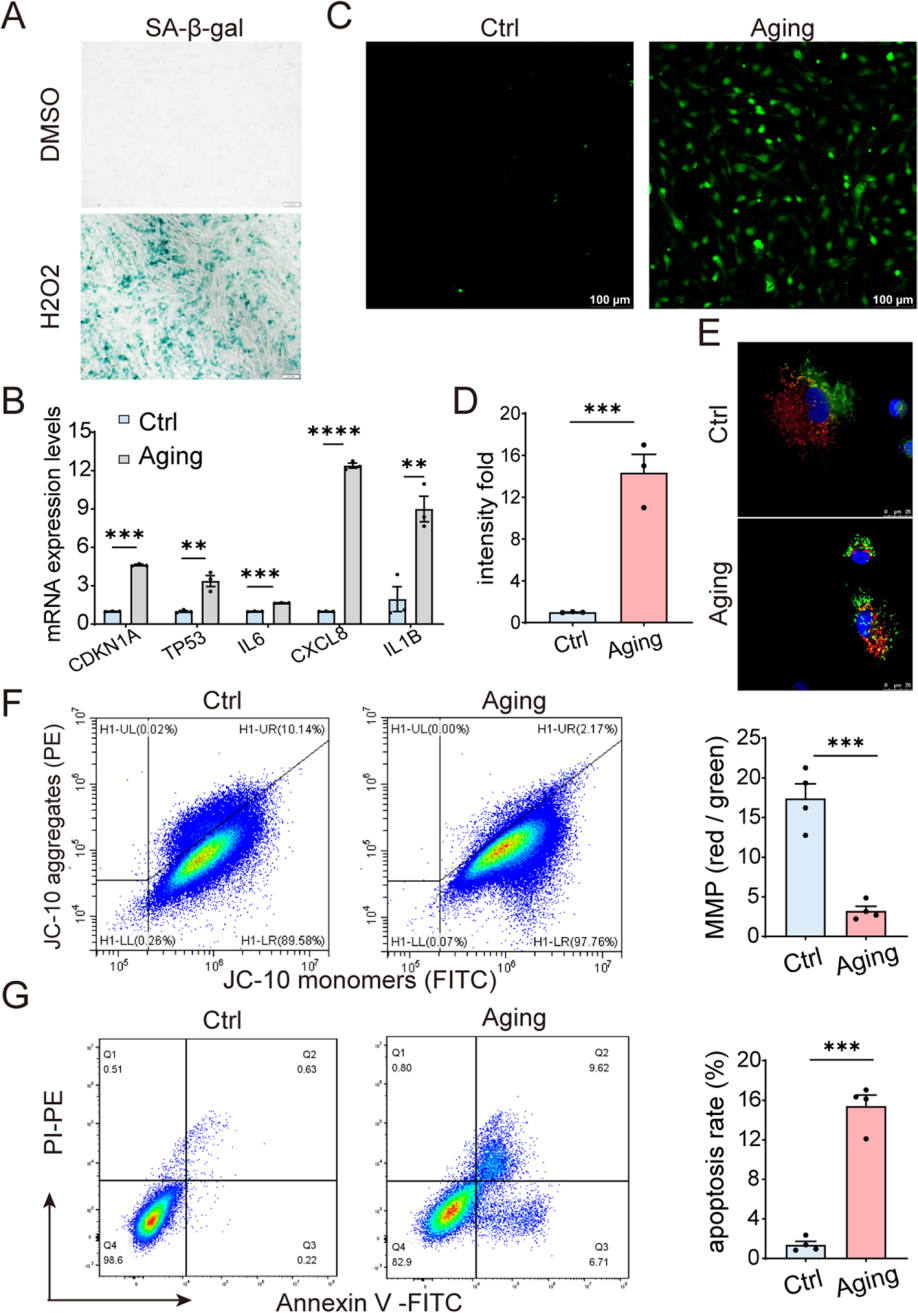
Enhanced oxidative stress and apoptosis in aging granulosa cells. **A** Senescent KGN were identified through SA-β-galactosidase (SA-β-gal) staining following treatment with H_2_O_2_ (300 µM, 4 h). **B** Evaluation of relative mRNA expression levels for genes associated with cellular senescence and the senescence-associated secretory phenotype (SASP) (*n* = 3 for each group). **C** Intracellular reactive oxygen species (ROS) levels were assessed after H_2_O_2_ treatment. Representative fluorescence images depicting aging KGN. **D** Quantitative analysis of DCFDA staining results (*n* = 3 for each group). **E** Measurement of mitochondrial membrane potential (MMP) by JC-10 staining after H_2_O_2_ treatment. Representative fluorescence images displaying aging KGN. JC-10 aggregates emit orange-red fluorescence while JC-10 monomers emit green fluorescence. **F** Flow cytometric assessment of MMP using flow cytometry and presentation of statistical data for MMP analysis (*n* = 4 for each group). **G** Annexin V and propidium iodide (PI) staining in KGN treated with H_2_O_2_ (300 µM, 4 h). And statistical representation of the apoptosis rate in senescent KGN (*n* = 4 for each group). Ctrl: control group. *P < 0.05, **P < 0.01, ***P < 0.001, ****P < 0.0001

Aging exerts multifaceted effects on cellular physiology, with significant consequences observed in various biological systems. Among these, GCs, critical regulators of ovarian function, have been subjected to extensive scrutiny in the context of aging-related changes. In cell aging system induced by H_2_O_2_, senescent KGN exhibit marked SA-β-gal activity (Fig. 3A). At the molecular level, there is an upregulation of senescence-associated secretory phenotype (SASP) factors (Fig. 3B), including *CDKN1A* (protein name: p21)*, TP53* (protein name: p53)*, IL6, CXCL8* (protein name: IL-8)*, IL-1β*.

Additionally, massive ROS production was detected in aged KGN (Fig. 3C-D), suggesting higher oxidative stress in aged KGN. Increased oxidative stress in senescent cells has been linked to the accumulation of dysfunctional mitochondria, manifested by the changes in mitochondrial mass, membrane potential and mitochondrial morphology [21]. As expected, the average mitochondrial membrane potential (MMP) in the aging GCs was significantly lower than that in the control group, indicating that GC senescence was accompanied with mitochondrial dysfunction (Fig. 3E, F). Furthermore, there were more apoptotic KGN in aging group compared with the control group (Fig. 3G).

### The ovarian immune milieu is altered with aging

**Fig. 4:**
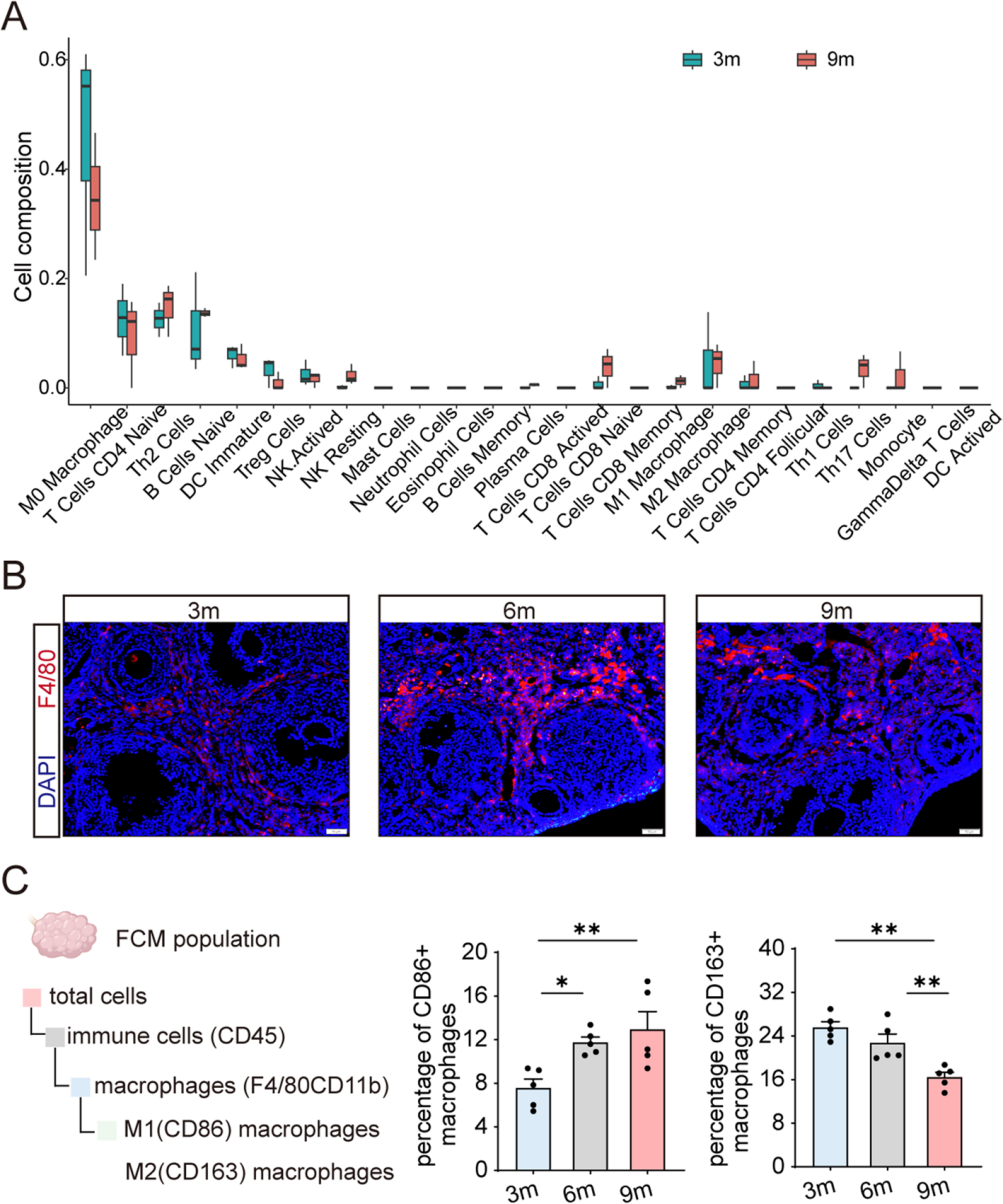
The ovarian immune milieu is altered with age. **A** Analysis of immune cell infiltration between ovarian tissues from mice at 9 months (9 m) and 3 months (3 m) of age (*Reproduction*, 2020). **B** Immunofluorescence staining of F4/80 in sections of ovarian tissue from mice. **C** The change of ovarian macrophages polarization over the course of aging. Percentage and number of M1 (CD86 +) and M2 (CD163 +) macrophage from the ovaries of mice at 3 months (3 m), 6 months (6 m), 9 months (9 m) of age were analyzed using flow cytometry (*n* = 5 for each group). The bar charts reflect the percentage of M1 (CD86 +) and M2 (CD163 +) macrophage within total ovarian macrophages. *P < 0.05, **P < 0.01, ***P < 0.001

The clearance of these cells represents a crucial process for maintaining homeostasis since the secretion of various cytokines by senescent cells can modify the microenvironment, with the immune system playing a prominent role [22]. We then investigated the disparity of immune cell infiltration and DEGs (Fig. S1A) between murine ovarian tissues at 3 months and 9 months [23]. As showed in Fig. 4A, considerable variations were evident in the composition of immune cells across the tissue samples and age groups in ovarian tissues. Notably, macrophages were the predominant infiltrating cell type (Fig. 4A). Among them, the most frequently observed decline was in M0 macrophages (Fig. 4A). At the protein level, cells expressing F4/80, a well-known macrophage marker [24], were specifically located in the theca of developing follicles at 3 months, which were potentially associated with ovulation. Of note, they were abundant in the interstitial tissue at 6 months (Fig. 4B).

Macrophage polarization serves as a candidate biomarker for assessing the inflammatory status and can be roughly classified into two types: pro-inflammatory classically activated macrophages (M1) and anti-inflammatory alternatively activated macrophages (M2) [15]. Here, a combination of CD45, F4/80 and CD11b was used to identify ovarian macrophages (Fig. 4C). Consequently, female mice aged 9 months exhibited elevated levels of the M1 marker (CD86) and reduced expression of the M2 marker (CD163) (Fig. 4C), indicating an age-related alteration in the ovarian immune milieu.

### Aging leads to the decline of ovarian macrophages phagocytosis

**Fig. 5:**
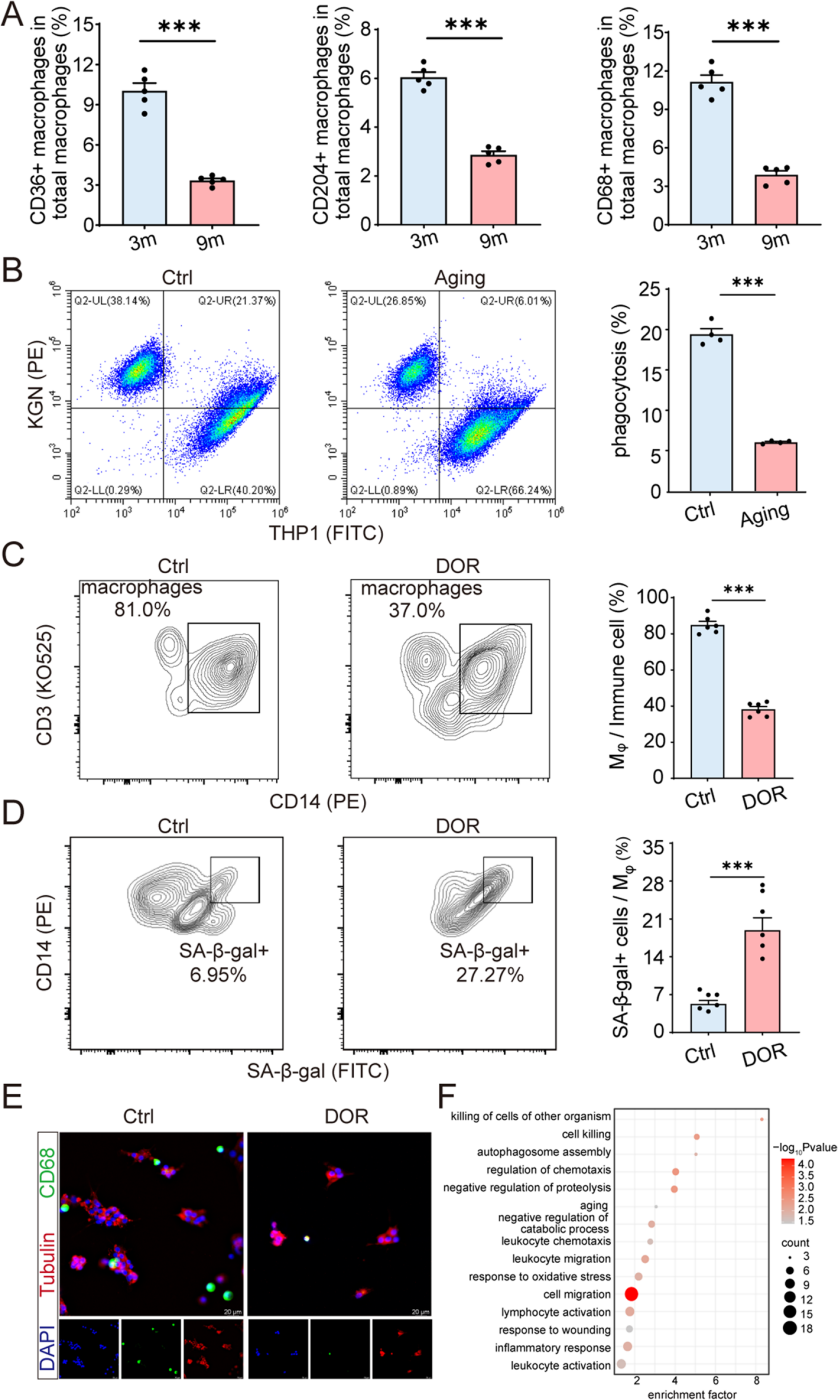
Aging-induced decline in ovarian macrophages phagocytosis. **A** Flow cytometry analysis was performed to assess the surface expression of scavenger receptors CD36, CD204, and CD68 on macrophages from the ovaries of mice at 3 months (3 m), and 9 months (9 m) of age. The resulting bar charts illustrate the proportion of phagocytic CD36^+^, CD204^+^, and CD68^+^ cells within the total macrophage population (CD11b^+^/F4/80^+^), with a sample size of *n* = 5 for each group. **B** H_2_O_2_-indecued aging macrophages (FITC) were incubated with aging KGN (PE) (at 1:2 ratio) for 3 h. Representative flow cytometry plots was presented and phagocytosis was analyzed by flow cytometry (*n* = 4, respectively). Ctrl: control group. **C** Representative flow cytometry plots illustrating the proportions of macrophages within immune cells populations in the follicular fluid (FF) of individuals with diminished ovarian reserve (DOR) and controls (Ctrl). Quantitative analysis of flow cytometry data, presenting the proportion of macrophages (Mφ) within the immune cell population (*n* = 6, respectively). **D** Representative flow cytometry plots illustrating the proportions of SA-β-gal^+^ cells within macrophages in the FF of DOR patients and Ctrl. Additionally, statistical analyses were conducted on these samples (*n* = 6 for each group). **E** Representative immunofluorescence images depicting CD68 expression within the FF of DOR and Ctrl individuals. F Gene Ontology (GO) enrichment analysis performed on biological processes between young (control group) and aging primate ovaries macrophages in the dataset GSE130664. *P < 0.05, **P < 0.01, ***P < 0.001

The DEGs between murine ovarian tissues at 3 months and 9 months were further processed for functional enrichment with Gene Ontology (GO) (Fig. S1B). The results showed that these DEGs are primarily involved in immune responses, including macrophage chemotaxis, cell killing, regulation of inflammatory response, phagocytosis, and macrophage migration. We further demonstrated a significant decrease in the expression levels of phagocytosis-associated molecules, including CD68, CD204, and CD36 in ovarian macrophages of the 9-month group, compared with 3-month group (Fig. S1C, Fig. 5A). Subsequently, the CRA003645 dataset was analyzed for double validation [25]. The heatmap provided a comprehensive overview of DEG patterns within mouse ovarian tissues across three distinct time points: 3 months, 6 months, and 9 months (Fig. S2A). Similarly, GO analysis conducted between 3-month and 9-month group revealed a plethora of DEG associated with phagocytosis (Fig. S2B). Consistent with findings from the 2020 cohort, we observed the decline in M0 macrophages within murine ovarian tissues of the 9-month (Fig. S2C).

To investigate the influence of aging on macrophage phagocytic activity, H_2_O_2_-induced macrophage aging was constructed in vitro, and evidenced by the substantial presence of SA-β-gal-positive cells in the aging group, as well as the elevated levels of SASP factors, including *CDKN1A*, *CDKN2A* (protein name: p16), *IL6, CXCL8*, and *IL-1β* (Fig. S3A-B). Notably, we observed a pronounced impairment in the phagocytic function of macrophages during aging, as indicated by a substantial reduction in the percentage of engulfed microspheres, declining from approximately 30% to 15% (Fig. S3C). More importantly, when using apoptotic human KGN as targets, a similar decrease in phagocytic capability of aging THP1 cells (human monocytic cell line) was observed (reduced from ~ 20% to ~ 5%) (Fig. 5B). After directly or indirectly co-culturing with aging KGN, there was a significant elevation in the SA-β-gal-positive THP1 in both conditions (Fig. S3D-E), indicating that aging GCs can accelerate the aging process in macrophages.

Subsequently, follicular fluids (FF) were collected from 10 patients with DOR and 10 control subjects. The age of these women ranged from 24 to 35 years, and their serum AMH levels ranged from 0.04 ng/mL to 7.59 ng/mL (Table 1). As shown, a significant decrease in the proportion of macrophages was observed in the DOR group, whereas the SA-β-gal-positive macrophages were notably increased (Fig. 5C, D). Immunostaining with CD68 revealed a less intense signal in the DOR patients compared to the control group (Fig. 5E). Additionally, the single-cell transcriptome data of ovaries from four young monkeys (4–5 years old, 1122 cells) and four aging (18–20 years old, 1479 cells) monkeys [26] were examined. GO analysis on the DEGs between aged and young primate ovarian macrophages demonstrated these DEGs were primarily involved in cell killing, autophagosome assembly, aging, cell migration, and inflammatory response (Fig. 5F).

**Table 1:** Clinical characteristics of diminished ovarian reserve (DOR)

Parameters	Ctrl (*n* = 10)	DOR (*n* = 10)	*P* value
Age (year)	30 (24–35)	31 (24–34)	0.5636
BMI (kg/m^2^)	22.17 (17.5–25.16)	22.00 (18.4–25.4)	0.9038
Basal serum FSH (mIU/mL)	6.078 (4.43–7.35)	15.23 (11.84–20.92)	0.0016
Basal serum E2 (pmol/L)	29.60 (17.80–44.00)	32.01 (21.5–44.48)	0.7574
Basal serum LH (mIU/mL)	5.15 (2.3–7.76)	4.19 (1.76–6.82)	0.3208
Basal serum T (nmol/L)	0.43 (0.27–0.65)	0.35 (0.12–0.51)	0.2724
Basal serum AMH (ng/mL)	4.94 (3.20–7.59)	0.80 (0.04–1.09)	< 0.0001
Antral follicle count (AFC)	12 (7–19)	4 (1–7)	0.0002

Collectively, the aged ovary exhibited impaired macrophage phagocytosis, likely influenced by aging granulosa cells, indicating intricate cellular dynamics with potential implications for overall ovarian health.

### Low molecular weight Chitosan (LMWC) alleviates ovary senescence via promoting macrophages phagocytosis

Chitosan, a polysaccharide derived from chitin, can be categorized into high, medium, or low molecular weight forms based on its molecular weight range [27]. Previous studies have reported that Chito-oligosaccharides (COS) enhance the phagocytic function of murine peritoneal macrophages following administration through peritoneal injection or intragastric delivery [28]. High-molecular-weight Chitosan (HMWC) has been demonstrated to induce anti-inflammatory polarization, enhance macrophage-released MMP9 activity, and promote migration in human monocyte-derived macrophages [29]. LMWC is distinguished by its heightened permeability, lower melting point, and increased water solubility in comparison to HMWC [30]. However, the specific impact of LMWC on ovarian macrophages and aging remains unexplored.

**Fig. 6:**
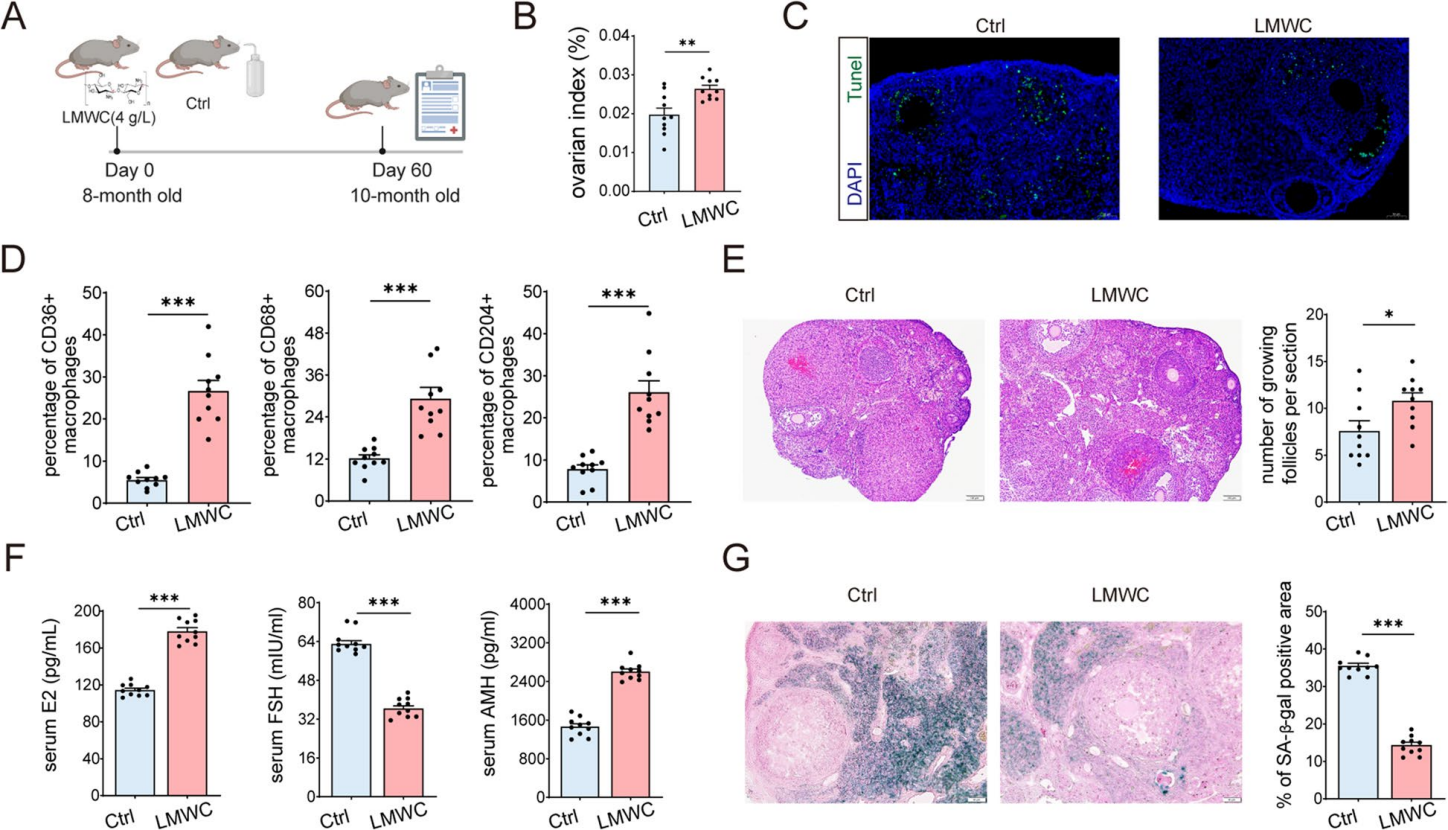
Low molecular weight Chitosan (LMWC) delays the ovary senescence via promoting phagocytosis of macrophages. **A** Eight-month-old mice were administrated LMWC in the drinking water (no LMWC as control) for 2 months in prevention experimental settings (*n* = 10 for each group). At the end point, mice were euthanized with the experimental mice to collect the ovaries for analysis. **B** Quantification of ovarian index (*n* = 10 for each group). **C** Representative images of TUNEL-labeled apoptotic cells (in green) with DAPI counterstaining (in blue) in the ovaries of mice. **D** Statistical analysis of the proportion of CD36^+^, CD204^+^, and CD68^+^ macrophages within the total macrophages, with a sample size of *n* = 10 for each group. **E** Histologic sections of representative ovaries from each treatment group, and average number of the growing follicles per section. **F** Quantification of serum levels of follicle-stimulating hormone (FSH), estradiol (E2), and anti-Müllerian hormone (AMH) obtained from LMWC-treated and control (Ctrl) mice (*n* = 10 for each group). **G** Representative images of senescence-associated β-galactosidase (SA-β-gal) staining in murine ovarian sections, and quantification of SA-β-gal positive area (*n* = 10 for each group). *P < 0.05, **P < 0.01, ***P < 0.001

Cell viability was calculated as previous [31]. Here, we found LMWC significantly enhanced viability in cell proliferation of THP-1 cells, especially at 4 mg/L (Fig. S4A). Treatment with 4 mg/L LMWC significantly increased the expression levels of CD36 and CD204 in PMA-induced M0 cells (Fig. S4B). Moreover, we also investigated in vivo and in vitro the effects of LMWC on macrophage polarization. LMWC treatment significantly enhanced the protein expression levels of CD80, CD163 and CD206 (Fig. S4C-D), inducing M0-like cells to exhibit the characteristics of M1 and M2 macrophages simultaneously. Administration of LMWC significantly increased the mRNA expression of *Nos2* (protein name: iNOS) and *Il10* (Fig. S4E), suggesting LMWC has a great potential to improve the polarization homeostasis of macrophages. The results of in vivo experiment revealed that LMWC did not affect the estrus cycle (Fig. 6A, Fig. S4F), significantly increased ovary weight and ovarian index (Fig. 6B, Fig. S4G). Remarkably, LMWC administration alleviated SASP-induced DNA damage and cell apoptosis in mouse ovary (Fig. 6C), indicating senescent cells accumulating in aging organs can be effectively cleared through this treatment. Correspondingly, LMWC treatment upregulated the expression levels of CD36, CD68, and CD204 on macrophages in mice (Fig. 6D). Moreover, LMWC-treated mice exhibited a higher count of growing follicles (Fig. 6E). Serum estradiol and AMH levels in the LMWC group were significantly higher compared to the control group, while the levels of FSH levels were lower than that of control (Fig. 6F). The presence of SA-β-gal positive areas in ovarian tissue was notably reduced in the LMWC group (Fig. 6G). These findings suggest that LMWC has the potential to enhance macrophage phagocytosis and phenotypes and further alleviate ovary senescence.

## Discussion

**Fig. 7:**
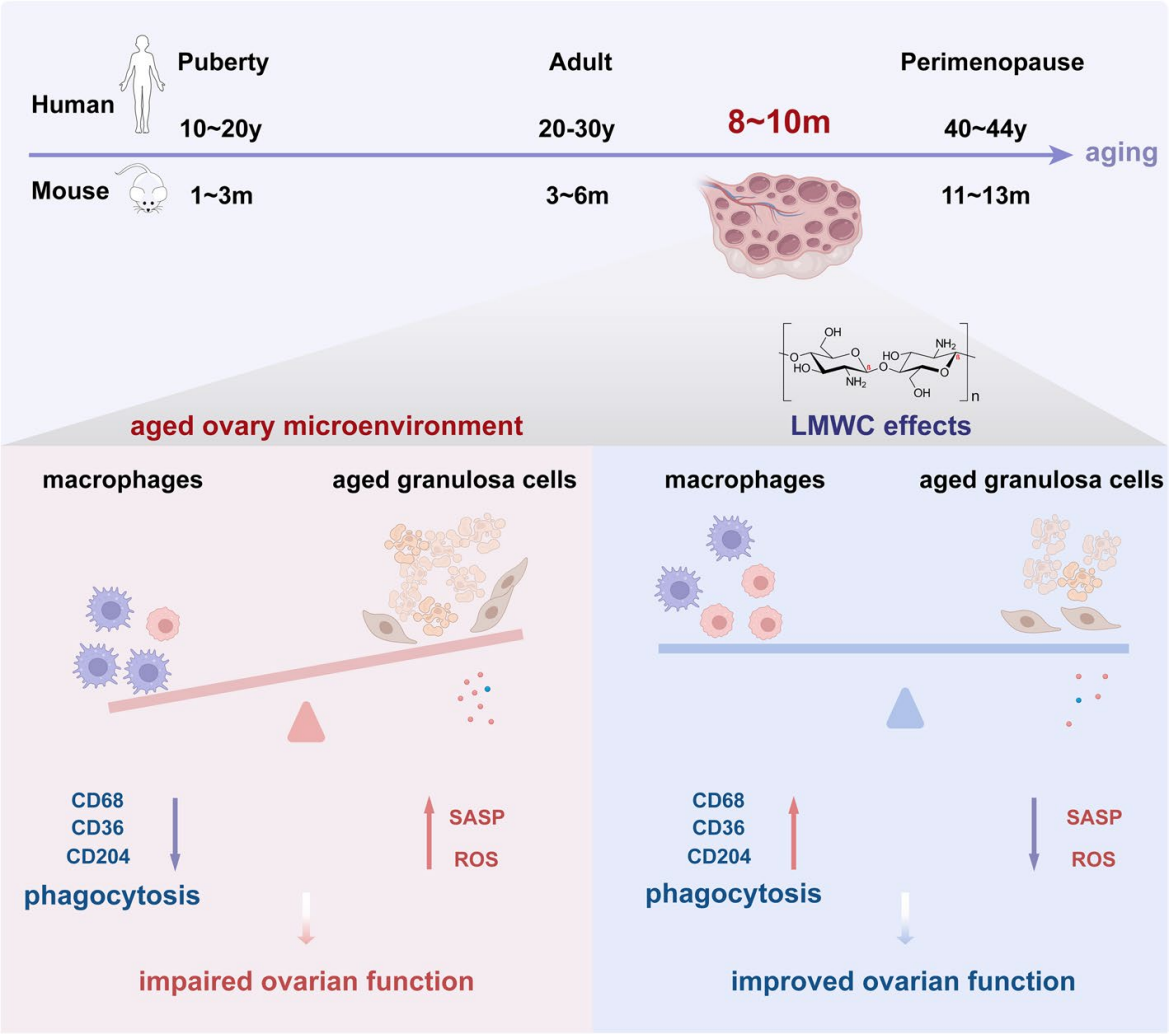
Chitosan alleviates ovarian aging by enhancing macrophage phagocyte-mediated tissue homeostasis. In this study, we found the aging ovarian macrophages exhibited insufficient expression of pivotal phagocytic macrophage markers, including CD36, CD204, and CD68. These diminished markers are indicative of impaired phagocytic capabilities in aging macrophages. Consequently, these aged cells tend to accumulate within the ovarian microenvironment, culminating in the release of a proinflammatory senescence-associated secretory phenotype (SASP). Due to the elevation of reactive oxygen species (ROS) within the local milieu, ovarian function was impaired. Following the administration of LMWC, a compelling and robust upregulation in the expression of CD36, CD204, and CD68, was observed within the ovarian macrophage population. In this way, LMWC is helpful for attenuating local SASP secretion and the production of ROS by enhancing macrophage phagocyte-mediated tissue homeostasis

In our study, we observed that the ovary aging was earlier than other organ systems in the C57BL/6 animal model. Mice at the age range of 3 to 9 months, were chosen to mirror the physiological reproductive phase, corresponding to the age range of 20 to 37 years in women (Fig. 7) [32]. This ovarian aging process will contribute to the diminishment of fertility and compromised endocrine functionality, thereby giving rise to a noteworthy societal concern, particularly in the context of the prevailing global trend towards delayed childbearing among women [33]. Thus, it is crucial to investigate the immunopathological mechanism underlying ovarian aging and explore potential interventions that should benefit the preservation of women's reproductive health and fertility.

Ovarian macrophages are essential for maintaining tissue homeostasis. Briley et al. found a stable quantity of F4/80-positive cells in both young and elderly CB6F1 mice [34]. However, they observed macrophage giant cells in older mice, absent in younger counterparts [34, 35]. Lliberos et al. noted an increase in CD4 + T cells, B cells, and macrophages during reproductive aging [36]. In humans, the percentage of CD68 cells is high in children (first and second decades) but tends to decrease with age [37]. Our study delved deeper into the role of ovarian macrophages and uncovered the dynamic changes in their activation state as the ovary ages.

There was increased follicular atresia with age and this process triggers apoptosis in a considerable number of cells [38]. Timely clearance of apoptotic cells is imperative for curbing inflammation [39]. Carlock et al. employed five phagocyte markers, including IA/IE (major histocompatibility complex class II), F4/80, CD11b (Mac-1), CD11c, and CD68 to investigate ovarian macrophage and/or phagocyte subpopulations and their distinct tissue distribution patterns [24]. A prior study revealed the accumulation of F4/80 macrophages around the senescent region of the implantation scar, where they participated in the clearance of postpartum uterine senescent cells [40]. More importantly, mice with uterine-specific p53 deletion showed the exacerbated retention of postpartum uterine senescent cells, the accumulation of CD11b cells, a decrease in F4/80 macrophages, a heightened senescence-associated inflammatory microenvironment, and a poor secondary pregnancy outcome [40]. Our study indicates a noteworthy reduction in the protein levels of CD36, CD68, and CD204 in the ovaries of 9-month mice compared to 3-month. Besides, KGN were exposed to H_2_O_2_ to induced oxidative stress in vitro*,* resulting in increased levels of SASP, ROS, and apoptotic rates. Similarly, aged macrophages exhibited a significant reduction in phagocytic ability. A recent study showed the clearance of senescent ovarian cells by immune cells was upregulated with aging to meet the ovarian requirements [41], possibly as a compensatory response to the diminished phagocytic capacity of aged macrophage. While Wong et al. reported a decline in alveolar macrophage counts, compromised phagocytosis of apoptotic neutrophils, and a decrease in the expression of the scavenging receptor CD204 with aging [42]. Our study revealed impaired macrophage phagocytosis in aged ovary was likely induced by the accumulation of aging GCs. The aged-related dysfunction in macrophage phagocytosis may be influenced by SASP in local microenvironment. The exact mechanism needs to be further researched.

Age represents a significant determinant in the attainment of successful pregnancies through IVF [43]. This is compounded by increased mean life expectancy that is not matched by women’s reproductive lifespan. Prior research reported higher mural GC apoptosis was associated with DOR, with fewer egg and embryo numbers in IVF/ICSI, as well as with age [44, 45]. Oxidative stress-induced apoptosis is recognized as a main primary factor contributing to follicular atresia [46, 47]. Our study exhibited an increased presence of senescent macrophages with impaired phagocytosis in the follicular fluid of patients with DOR. This might contribute to the altered ovarian microenvironment seen in DOR conditions, potentially affecting the ovarian tissue’s ability to maintain homeostasis and proper function.

COS is the degraded products of chitin/chitosan by acid hydrolysis, enzymatic degradation or both [48]. It has been reported that COS and macrophages have synergistic effects on improving OGSCs function by regulating inflammatory factors [28]. Additionally, COS protected GCs from H_2_O_2_-stimulated oxidative damage and apoptosis by inactivating the HIF-1α-VEGF signaling pathway [49]. In line with this, a recent research highlights COS’s potential in alleviating inflammation and oxidative stress of ovarian GCs from patients with PCOS [50]. In this study, we found LMWC significantly enhanced macrophage proliferation, immunomodulation, and phagocytic activity, thereby improving the ovarian microenvironment. LMWC induced and increased in both M1 and M2 marker expressions in macrophages, indicating its potential to elicit a multifaceted immune response. This dual modulation is particularly significant in the ovarian immune microenvironment. The ovary, a dynamic organ, is regulated by hormones but also by the surrounding immune context. Situations like ovarian inflammation or aging necessitate a nuanced approach: the ovarian tissue may need a pro-inflammatory response for the clearance of damaged cells and pathogens, alongside an anti-inflammatory or reparative response to foster tissue recovery and sustain normal functionality. Previous research has also highlighted the improvement in phenotype homeostasis of macrophages by chitosan nanoparticles and therapeutic impacts on liver injury [51] and skin area repair [52]. Moreover, the molecular weight of chitosan, particle size, and crosslink degrees influenced only the efficiency but not the trend of immunoregulatory effects of chitosan nanoparticles [51]. Crucially, our findings suggest that LMWC could improve macrophage-mediated clearance of ovarian senescent cells, offering a potential treatment strategy for preserving and reshaping ovarian function. However, there are still some limitations. The scarcity of total ovarian macrophage counts hindered our ability to isolate the sufficient quantity of ovarian macrophage for comprehensive analysis. Moreover, the specific mechanisms underlying the decline in macrophage phagocytic function associated with aging remain to be further elucidate.

## Conclusions

In conclusion, we elucidated advanced reproductive age appears to be associated with impaired macrophage phagocytic function and aging GCs, indicating a positive correlation between macrophage phagocytic dysfunction and DOR during ovarian aging. LMWC can enhance macrophage phagocytosis and further alleviate ovarian aging.

## Methods

### Human follicular fluid (FF) collection

The follicular fluid (FF) was obtained from women undergoing oocyte retrieval and intracytoplasmic sperm injection (ICSI) at the Reproduction and Genetics Centre of the Affiliated Suzhou Hospital of Nanjing Medical University. Ten women diagnosed with DOR patients (age: 31 ± 3.94 y [mean ± *SD*]) and ten healthy controls (age: 30 ± 3.85 y [mean ± *SD*]) were recruited in this study from March 1st, 2023 to June 1st, 2023, matched according to body mass index and age. Informed consent was signed by all study participants. The diagnosis of DOR was based on the presence of FSH levels > 10 IU/L, AMH levels < 1.1 ng/ml, and antral follicle count (AFC) < 7 [53]. Individuals with the following conditions were excluded from the study: (i) endometriosis, polycystic ovarian syndrome (PCOS), and premature ovarian insufficiency; (ii) chromosomal abnormalities; (iii) hypertension, diabetes, or other chronic diseases; (iv) immune system diseases, such as hypothyroidism. This study was performed under institutional review board approval.

### Animals

Female C57BL/6 mice were purchased from Cyagen Company (Shanghai, China). The weight of each extracted ovary was promptly recorded upon removal. The ovarian index was calculated as the ratio of ovary weight to body weight.

A solution was prepared by rehydrating 1.2 g of low molecular weight Chitosan (LMWC) in 300 ml drinking water. To investigate the impact of LMWC supplementation, eight-month-old female mice were assigned randomly into two groups. The control group received drinking water, while the LMWC group was administered LMWC at a concentration of 4 g/L dissolved in their drinking water for a duration of 2 months. The estrous cycles of the female mice were assessed daily by vaginal smears for 14 days.

### Senescence-associated beta-galactosidase (SA-β-gal) staining

Tissue samples were o fixed in a 4% paraformaldehyde (PFA) solution. According to the manufacturer’s protocol, the fixed tissue cryo-sections or cells were then incubated with SA-β-gal staining kit (C0602, Beyotime) at 37°C overnight, in the absence of CO_2_. After washed twice with phosphate-buffered saline (PBS), the sections were counterstained for 30 s in eosin and washed twice with PBS. Senescent cells and tissues were identified as blue-green-stained under an inverted microscope.

### Immunohistochemistry (IHC)

Tissue sections were treated with blocking solution for 30 min. Afterwards, the sections underwent an overnight incubation at 4 °C with primary antibodies targeting p16 (sc-1661, 1:500, Santa Cruz), and p21 (sc-397, 1:500, Santa Cruz). Following three washes with PBS, the sections was stained with 3,3-diaminobiphenylamine (DAB, Sigma) and hematoxylin (Beyotime). Finally, images of the stained sections were acquired utilizing an Olympus BX51 fluorescence microscope (Tokyo, Japan).

### Apoptosis assay

Human granulosa cell lines (KGN) were cultured in 12-well plates and treated with H_2_O_2_ (300 µM) for 4 h to induce senescence. Apoptosis was assessed by flow cytometry after staining with fluorescein-labeled annexin V and propidium iodide (PI) (FITC Annexin V Apoptosis Detection Kit with PI, C1062, Beyotime, China) for 30 min at room temperature in the dark.

TUNEL assay performed by using a commercial TUNEL apoptosis assay kit (C1086, Beyotime, China) following the manufacturer’s instructions. The frozen slides were stained with TUNEL reaction mixture in the dark at 37 °C for 1 h. Subsequently, the slides were stained using DAPI (C1005, Beyotime, China) staining solution. The slides were mounted on coverslips using anti-fade mounting media (ab104135, Abcam) and then captured under a fluorescence microscope.

### Histological examinations and follicle counts

Ovary sections were stained using hematoxylin–eosin solution (HE, C0105S, Beyotime) according to the instructions. Images were taken with a light microscope. The evaluation of ovarian follicles was performed by two senior experts using a light microscope. Primordial, primary, secondary and tertiary follicles were classified according to the corresponding histological morphology [54]. Five times the count value was taken as the result.

### Real-time quantitative polymerase chain reaction (RT-qPCR)

Total RNA containing cells and ovary tissue were extracted by Trizol method. The RNA was reverse-transcribed according to the instructions of kit (Cat# 11141ES10, Yeasen, Shanghai, China,). RT-qPCR was implemented using Hieff UNICON® Universal Blue qPCR SYBR Master Mix (Cat# 11184ES03, Yeasen) on a Quant Studio 6 Flex RT-PCR System. The primer are as follows in Supplementary Table 1.

### Immunofluorescence staining

Following fixation, the slides were permeabilized using 0.1% Triton-X for 20 min. Subsequently, the slides were placed in a blocking buffer containing 10% goat serum and 1% BSA in PBS for 1 h. Next, the sections were incubated with primary antibody (cat# 41–4801-80, F4/80, Invitrogen) overnight at 4 °C. After washing with PBS, they were incubated with secondary antibody for 1 h at room temperature and then stained with DAPI solution for 10 min.

### Detection of reactive oxygen species (ROS)

The staining procedure was based on a reported protocol [31]. In brief, cells were cultured 5 × 10^4^ cells per well of 12-well plate in 1 ml of complete medium for 24 h. For measurement of the effect of H_2_O_2_ treatment on ROS generation, the wells were washed with PBS after H_2_O_2_ treatment, loaded with 5 μM DCFH-DA solution in PBS in the dark for 20 min at 37 °C. Then the medium was removed, cells were washed with PBS, and maintained in PBS. Finally, fluorescence microscopy (Thunder image) was used to detect and collect images.

### Mitochondrial Membrane Potential (MMP) Assay

To measure mitochondrial membrane potential (MMP), KGN were incubated with the fluorescent dye JC-10 Mitochondrial Membrane Potential Kit (Cat#421902, Biolegend, USA). The ratio of red (585 nm) to green (530 nm) fluorescence represents the changes in mitochondrial membrane potential. KGN were stained with JC-10 for 15 min at 37 °C after treatment with 300 μM H_2_O_2_ for 4 h. JC-10 fluorescence was detected using flow cytometry. Meanwhile, they were also stained with DAPI and imaged immediately in both green and red channels using excitation at 488 nm and emission at 530 nm (green signals) together with excitation at 568 nm and emission at 590 nm (red signals) under a laser scanning confocal microscope. At low concentrations (due to low MMP), JC-10 is predominantly a monomer that yields green fluorescence whereas, at high concentrations (due to high MMP), the dye aggregates yielding a red to orange color.

### Flow cytometry (FCM)

Cells washed twice and suspended in 1 ml 0.5% BSA-PBS. Following preincubation with mouse or human Fc block (cat#422,302, Biolegend, USA), the cell suspension was stained with antibodies (Supplementary Table 2) at 25 °C for 30 min in the dark. Following a series of washes and resuspension in PBS, the cells were subjected to analysis using a flow cytometer.

### Phagocytosis assay

PMA-treated THP-1 were detached using trypsin and resuspended in 100 μl PBS. To each cell suspension tube, add 1 μL each of anti-human CD45 (APC) and FITC-microsphere (both sourced from Biolegend) for phagocytosis analysis. Gentle vortex each tube and incubate in the dark at room temperature for 30 min. Subsequently, cells were analyzed by FCM.

To assess cell phagocytosis through the quantification of KGN engulfment, young or aged KGN were stained with PKH26 (PE), following the manufacturer's guidelines (Sigma-Aldrich, St. Louis, MO, USA). Briefly, 10 µl of KGN from pellet was diluted in 125 µl diluent C (supplied with the PKH26 kit), added to 0.5 µl PKH26 in 125 µl diluent C. The mixture was immediately incubated at room temperature for 4 min and then terminated by the addition of 10 ml of complete culture medium. PMA-differentiated THP-1 cells were stained with PKH27 (FITC). KGN and PMA-treated THP1s were co-incubated at 37 °C for 3 h. The data were analyzed using FlowJo software, and the percentage of macrophages strongly positive for PKH26-red-labelled KGN, relative to the control group, was calculated.

### Transcriptomics analysis

RNA-seq data were obtained from two sources: https://bigd.big.ac.cn/gsa (CRA003645) [25] and supplementary material [23]. To analyze the differentially expressed genes (DEGs), the limma software [55] were used, based on a fold change criterion (|logFC|> 1) and a significance threshold (*P*-value < 0.05). The resultant DEGs were visualized through a volcano plot using the R package "ggplot2" [56] and a heatmap employing the R package "ComplexHeatmap" [57].

The CIBERSORT algorithm [58], was used for estimating the relative proportion of immune cells. For GO term enrichment analysis of DEGs, the R package "ClusterProfiler" [59] were employed. Enriched GO terms with a significance level of *P* < 0.05 were considered statistically significant.

### Statistical analysis

The statistical analysis was conducted using GraphPad Prism software (v8.0). When the data exhibited normal distribution, students’ t-test was used to compare two groups, and one-way ANOVA followed by Bonferroni's test was employed for multiple groups. In the absence of normal distribution, either the Mann–Whitney U test or the Kruskal–Wallis test was utilized. All data were presented as mean ± *SEM*, and a *P* value less than 0.05 was considered statistically significant.

### Supplementary Information

**Additional file 1: Supplementary Table 1.** Gene primers for qRT-PCR. **Supplementary Table 2.** Antibodies for flow cytometry. **Fig. S1.** Volcano plot and gene ontology (GO) pathway analysis of differentially expressed genes (DEG) and flow cytometry gating strategy. **Fig. S2.** Differentially expressed genes (DEGs) analysis from the CRA003645 dataset. **Fig. S3.** Impaired phagocytosis in senescent macrophages influenced by aging KGN.

## Data Availability

All data associated with this study are available in the main text or the Supplementary Materials.

## Abbreviations

AFC: Antral follicle count
AMH: Anti-Müllerian hormone
BSA: Bovine serum albumin
DOR: Diminished ovarian reserve
DEGs: Differentially expressed genes
FCM: Flow cytometry
FF: Follicular fluid
FSH: Follicle-stimulating hormone
GO: Gene ontology
HE: Hematoxylin–eosin
HMWC: High-molecular-weight Chitosan
LMWC: Low-molecular-weight Chitosan
ICSI: Intracytoplasmic sperm injection
IVF: In-vitro fertilization
IHC: Immunohistochemistry
LH: Luteinizing hormone
MMP: Mitochondrial membrane potential
PCOS: Polycystic ovarian syndrome
PFA: Paraformaldehyde
PI: Propidium iodide
p16: Cyclin-dependent kinase inhibitor 2A
p21: Cyclin-dependent kinase inhibitor 1A
PBS: Phosphate-buffered saline
ROS: Reactive oxygen species
SASP: Senescence-associated secretory phenotype
