# Supplementary material for: Chitosan alleviates ovarian aging by enhancing macrophage phagocyte-mediated tissue homeostasis
Source: Immun Ageing. 2024 Jan 27;21:10. doi: 10.1186/s12979-024-00412-9 (PMC10821576; doi:10.1186/s12979-024-00412-9)
Supplement: Supplementary file 1 — Additional file 1: Supplementary Table 1. Gene primers for qRT-PCR. Supplementary Table 2. Antibodies for flow cytometry. Fig. S1. Volcano plot and gene ontology (GO) pathway analysis of differentially expressed genes (DEG) and flow cytometry gating strategy. Fig. S2. Differentially expressed genes (DEGs) analysis from the CRA003645 dataset. Fig. S3. Impaired phagocytosis in senescent macrophages influenced by aging KGN. [file 12979_2024_412_MOESM1_ESM.pdf]

Supplementary information

Supplementary Table 1. Gene primers for qRT-PCR

Supplementary Table 2. Antibodies for flow cytometry

Fig. S1. Volcano plot and gene ontology (GO) pathway analysis of differentially expressed genes (DEG) and flow cytometry gating strategy.

Fig. S2. Differentially expressed genes (DEGs) analysis from the CRA003645 dataset.

Fig. S3. Impaired phagocytosis in senescent macrophages influenced by aging KGN.

Supplementary Table 1. Gene primers for qRT-PCR

Tnfa (mouse)	Forward	CTGAACTTCGGGGTGATCGG
	Reverse	GGCTTGTCACTCGAATTTTGAGA
Il10 (mouse)	Forward	GCTGGACAACATACTGCTAACC
	Reverse	ATTTCCGATAAGGCTTGGCAA
Il6 (mouse)	Forward	GGTGCCCTGCCAGTATTCTC
	Reverse	GGCTCCCAACACAGGATGA
Il18 (mouse)	Forward	GTGAACCCCAGACCAGACTG
	Reverse	AACCCTCTGCACCCAGTTTTC
Il1b (mouse)	Forward	CCTGGAACACGTTTCTGAAAGA
	Reverse	TGGATGCTCTCATCAGGACAG
Nos2 (mouse)	Forward	GTTCTCAGCCCAACAATACAAGA
	Reverse	GTGGACGGGTCGATGTCAC
Actb (mouse)	Forward	GGCTGTATTCCCCTCCATCG
	Reverse	CCAGTTGGTAACAATGCCATGT
CDKN2A (human)	Forward	ATGGAGCCTTCGGCTGACT
	Reverse	GTAACTATTCGGTGCGTTGGG
CDKN1A (human)	Forward	CGATGGAACTTCGACTTTGTCA
	Reverse	GCACAAGGGTACAAGACAGTG
TP53 (human)	Forward	GAGGTTGGCTCTGACTGTACC
	Reverse	TCCGTCCCAGTAGATTACCAC
IL6	Forward	ACTCACCTCTTCAGAACGAATTG

(human)	Reverse	CCATCTTTGGAAGGTTCAGGTTG
CXCL8	Forward	ACTGAGAGTGATTGAGAGTGGAC
(human)	Reverse	AACCCTCTGCACCCAGTTTTC
IL1B	Forward	ATGATGGCTTATTACAGTGGCAA
(human)	Reverse	GTCGGAGATTCGTAGCTGGA
ACTB	Forward	GGCATCCTCACCCTGAAGTA
(human)	Reverse	TAGCACAGCCTGGATAGCAA

Supplementary Table 2. Antibodies for flow cytometry

Antibody	Clone	Fluorochrome	Species reactivity
Anti-CD45	30-F11	APC/Fire™ 750	Mouse
Anti-F4/80	BM8	FITC	Mouse
Anti-CD11b	M1/70	APC	Mouse
Anti-CD163	S15049I	PE	Mouse
Anti-CD86	PO3	Violet 421	Mouse
Anti-CD36	HM36	PE/Cyanine7	Mouse
Anti-CD204	1F8C33	PE	Mouse
Anti-CD68	FA-11	Violet421	Mouse
Anti-CD45	2D1	Violet 605	Human
Anti-CD206	15-2	PE/Cyanine7	Human
Anti-CD14	HCD14	FITC	Human

Anti-CD163	GHI/61	Violet 421	Human
Anti-CD80	2D10	PE/Cyanine5	Human
Anti-CD86	BU63	APC	Human
Anti-CD14	M5E2	PE	Human
Anti-CD3	OKT3	Violet 510	Human
Anti-CD68	Y1/82A	APC	Human

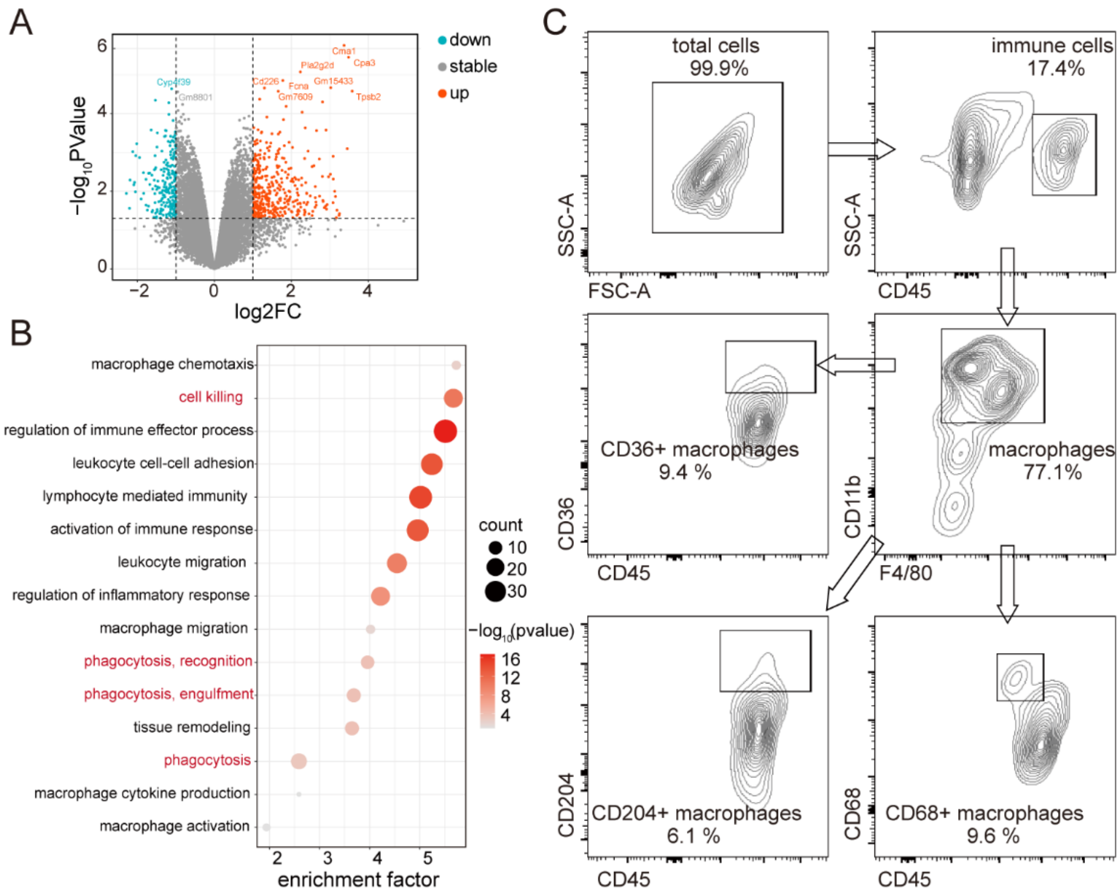

Fig. S1. Volcano plot and gene ontology (GO) pathway analysis of differentially expressed genes (DEG) and flow cytometry gating strategy.

A) Volcano plot representation of differential gene expression between 9-month (9m) and 3-month (3m) ovarian tissues.

B) Enriched biological processes identified through Gene Ontology (GO) analysis.

C) Schematic representation of representative gating strategies employed in the flow cytometry analysis.

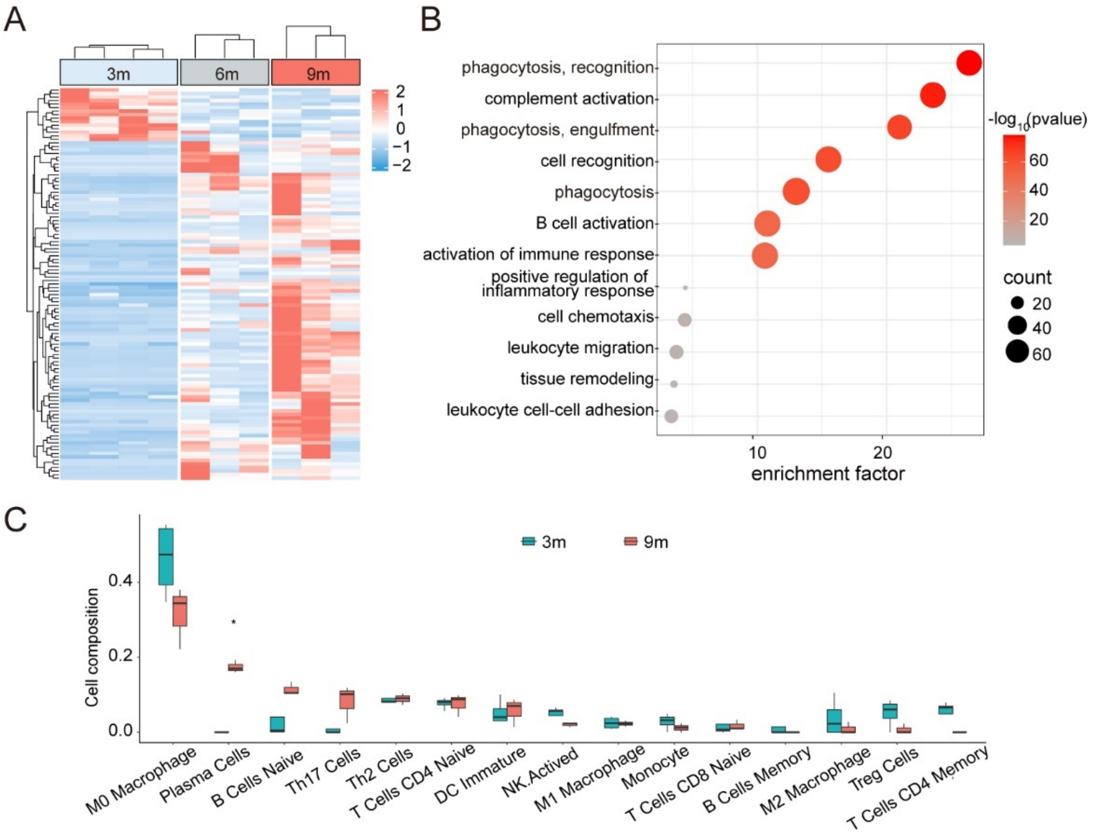

Fig. S2. Differentially expressed genes (DEGs) analysis from the CRA003645 dataset.

A) Heatmap result of an unsupervised hierarchical clustering of genes that is significantly different ($P < 0.05$) expressed within mouse ovarian tissues collected from mice aged 3 months (3m), 6 months (6m), and 9 months (9m) in the dataset CRA003645, accessible at https://bigd.big.ac.cn/gsa.

B) Gene ontology (GO) enrichment analysis conducted on the biological processes associated with DEGs between the ovaries of mice at 3 months and 9 months of age within the dataset CRA003645.

C) Analysis of immune cell infiltration levels based on CIBERSORT within ovarian tissues obtained from mice aged 9 months and 3 months in the dataset CRA003645.

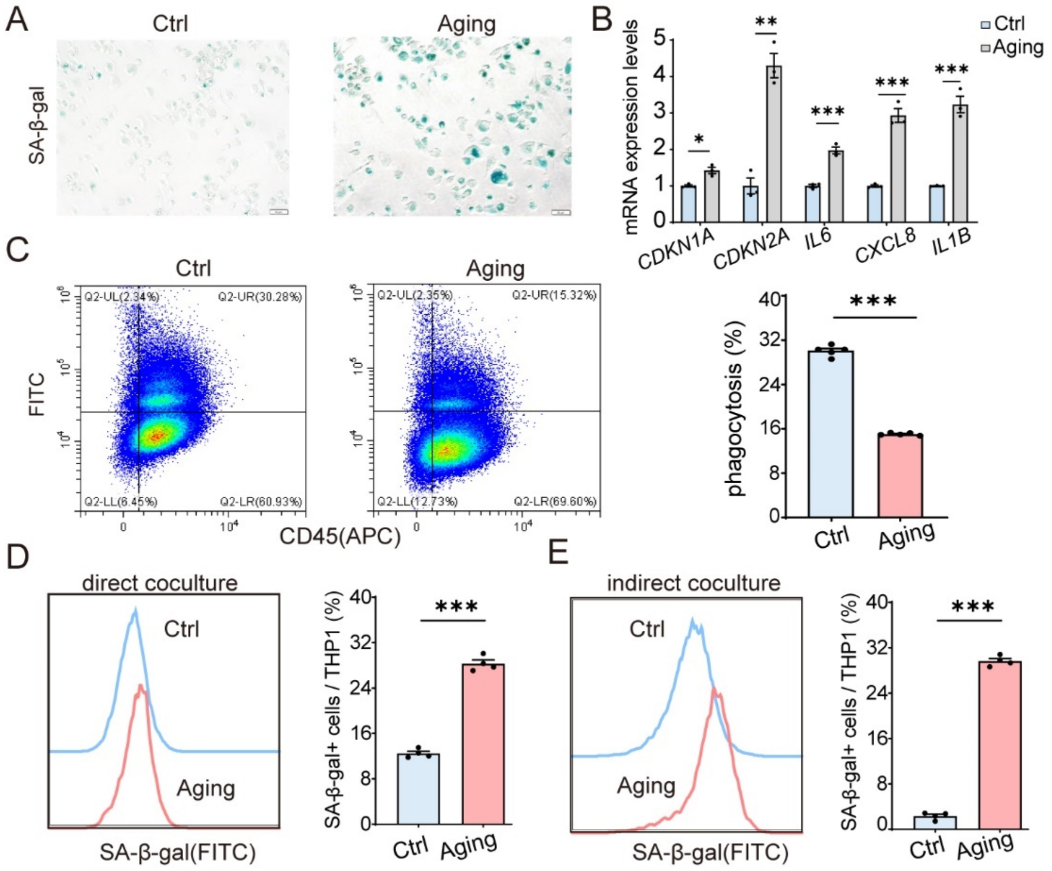

Fig. S3. Impaired phagocytosis in senescent macrophages influenced by aging KGN.

A) Senescent macrophages (THP1) were identified through SA-β-galactosidase (SA-β-gal) staining following treatment with H_2O_2 (300 μM, 36h).

B) Evaluation of relative mRNA expression levels for genes associated with cellular senescence and the senescence-associated secretory phenotype (SASP) (n = 3, respectively).

C) Evaluation of phagocytic activity in H_2O_2-induced senescent macrophages (APC) incubated with fluorescent particles (FITC) (50 particles/cell) for 2 hours. Representative flow cytometry plots are presented, and phagocytosis was quantified (n = 5, respectively).

D) Direct coculture of H_2O_2-induced senescent KGN with THP1 macrophages, followed by assessment of SA-β-gal$^+$ cells in macrophages. Overlay histogram demonstrates the shift in fluorescence of cells (n = 3, respectively).

E) Indirect coculture of H_2O_2-induced senescent KGN with THP1 macrophages, followed by assessment of SA-β-gal$^+$ cells in macrophages. Overlay histogram demonstrates the shift in fluorescence of cells (*n* = 3, respectively).

Ctrl: control group. $*P < 0.05$, $**P < 0.01$, $***P < 0.001$

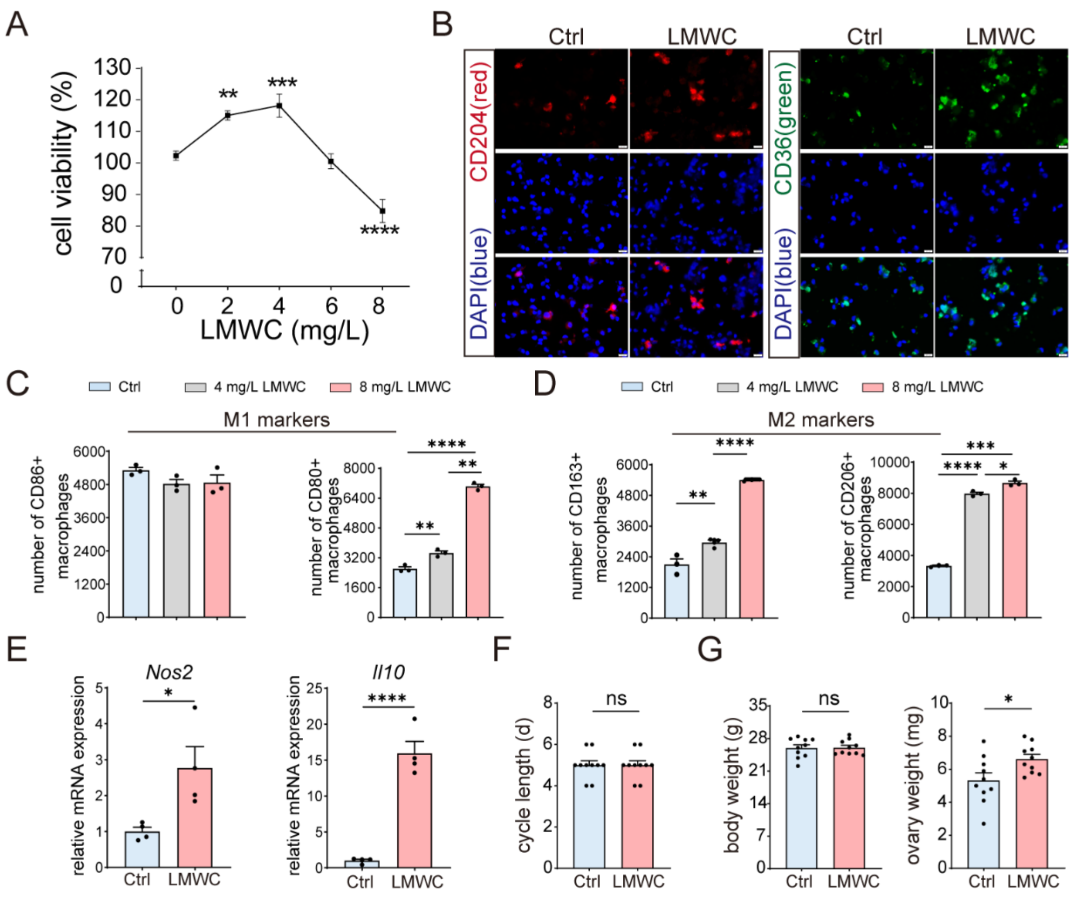

Fig. S4. Low molecular weight Chitosan (LMWC) improves the phenotype homeostasis of macrophage.

A) Assessment of cell viability using the cell counting kit-8 (CCK8) assay.

B) Immunofluorescence staining depicting CD36 (in green) and CD204 (in red) expression in PMA-differentiated THP-1 macrophages (M0, Ctrl) exposed to 4 mg/L LMWC for 48h.

C) THP-1 cells differentiated in PMA (100 ng/mL, 48h) for 2 days. The PMA-differentiated THP-1 macrophages (M0, Ctrl) were exposed to 4 mg/L LMWC or 8 mg/L LMWC for 48h (*n* = 3, respectively). Flow cytometry analysis of expression of M1 surface markers (CD80 and CD86) in $1*10^5$ M0 cells.

D) Flow cytometry analysis of expression of M2 markers (CD163 and CD206) in $1*10^5$ M0

cells.

E) LMWC treatment did not impact estrous cycles, and the cycle lengths between the two groups were comparable (*n* = 10, respectively).

F) Comprehensive data on body weight, ovary weight in the LMWC-treated group and control (Ctrl) group (*n* = 10, respectively).

G) The relative mRNA expression levels of *Nos2* (M1 phenotype, protein name: INOS) and *Il10* (M2 phenotype) in LMWC-treated mouse ovaries and control (Ctrl) groups (*n* = 4, respectively).

Data are presented as mean ± *SEM*. $^{*}P < 0.05$, $^{**}P < 0.01$, $^{***}P < 0.001$
